# Preconception care knowledge and information delivery modes among adolescent girls and women: a scoping review

**DOI:** 10.4069/kjwhn.2023.02.28

**Published:** 2023-03-31

**Authors:** Wiwit Kurniawati, Yati Afiyanti, Lina Anisa Nasution, Dyah Juliastuti

**Affiliations:** 1 Department of Maternity and Women Health, Faculty of Nursing, Universitas Indonesia, Depok, West Java, Indonesia; 2 Program Study of Nursing, Faculty of Sport and Health Education, Universitas Pendidikan Indonesia, Bandung, Indonesia; 3 Nursing Program, Sekolah Tinggi Ilmu Kesehatan Ichsan Medical Centre Bintaro, Tangerang Selatan, Indonesia

**Keywords:** Adolescent, Adult, Female, Pregnancy, Preconception care

## Abstract

**Purpose:**

The aim of this study was to conduct a scoping review of knowledge and information delivery modes related to preconception care (PCC) among adolescent girls and women.

**Methods:**

A scoping review was performed on studies selected from five electronic databases (Cochrane Library, PubMed, Science Direct, CINAHL/EBSCO, and ProQuest), published between 2012 and 2022, with predetermined keywords and criteria. We included English-language research articles available in full text and excluded irrelevant articles.

**Results:**

This study included eight articles, comprising seven quantitative studies and one qualitative study conducted among adolescent girls and women. Five were from low- and middle-income countries and three were from high-income countries. The synthesized themes generated from the data were PCC knowledge and PCC information delivery modes and effectiveness. In general, adolescent girls and women were found to have basic PCC knowledge, including risk prevention and management and a healthy lifestyle, although more extensive knowledge was found in higher-income countries than in lower-income countries. The delivery modes of PCC information have grown from individual face-to-face conventional methods, which are used predominantly in lower-income countries, to more effective digital mass media.

**Conclusion:**

Globally, many women still have insufficient knowledge regarding PCC, as not all of them receive access to PCC information and support. PCC promotion efforts should be initiated earlier by involving a wider group of reproductive-age women and combining individual, in-group, face-to-face, and electronic delivery modes.

## Introduction

Preconception care (PCC) has been a focus of attention since the Millennium Development Goals program and continuing into the Sustainable Development Goals program, which aims to optimize maternal health before conception. Many developed and developing countries have established PCC as an important health initiative [1,2]. This preconception agenda reflects the long-term goal of reducing maternal and infant mortality rates, which have recently been a paramount issue in the health sector [3,4]. PCC encompasses behavioral, biomedical, and social interventions performed by women and their partners before conception to identify health problems, behaviors that may lead to health issues, and personal and environmental risk factors contributing to maternal and infant mortality and morbidity [5,6].

Various internal and external obstacles can deter the implementation of PCC. Client-related barriers include unawareness of PCC services, unwillingness to participate in PCC programs, and religious beliefs [7]. Meanwhile, a lack of support from healthcare services, such as insufficient preparation for health promotion [7,8], low self-efficacy of health providers in conveying the programs [6-8], and limited environmental context and resources [7,8], may decrease women’s interest in using PCC services. These conditions require an exceptional level of understanding since the topic of PCC is still underdeveloped [7,8]. In the era of digitization, modifying the delivery of health campaigns and communication through web applications and social media has become an essential and convenient solution to increase awareness of reproductive health and access to health services [9,10].

The content of PCC education is extensive, including nutritional deficiencies, smoking behavior, and the potential health consequences of such high-risk behaviors. Moreover, it covers the risks of maternal and fetal illnesses related to environmental factors, genetic disorders, unwanted pregnancy, sexually transmitted infections, infertility, psychiatric disorders, substance and drug abuse, and violence issues [5,6]. Successful PCC implementation is attributed to a multisectoral program for healthy pregnancy targeting girls and women and involving families, schools, community platforms, and healthcare institutions [1,5]. Nevertheless, limited PCC programs tailored to adolescent girls and women have been implemented in low and middle-income countries [1]. The targets of PCC include adolescent girls and women who have not yet conceived and are in the process of getting married. As PCC knowledge and practices have been explored quite intensely in recent years, we conducted this study to answer the following research question: “According to the existing literature, what is known about the scope of essential PCC knowledge and informational delivery modes among adolescent girls and women?”

## Methods

We conducted a scoping review to summarize the research literature regarding essential PCC knowledge and informational delivery modes among adolescent girls and women across nations with different economic backgrounds. This review adhered to the PRISMA-ScR (Preferred Reporting Items for Systematic Reviews and Meta-Analyses for Scoping Reviews) [11], which guided the authors in developing a scoping report to present the findings.

### Search strategy and study selection

**Figure 1.**
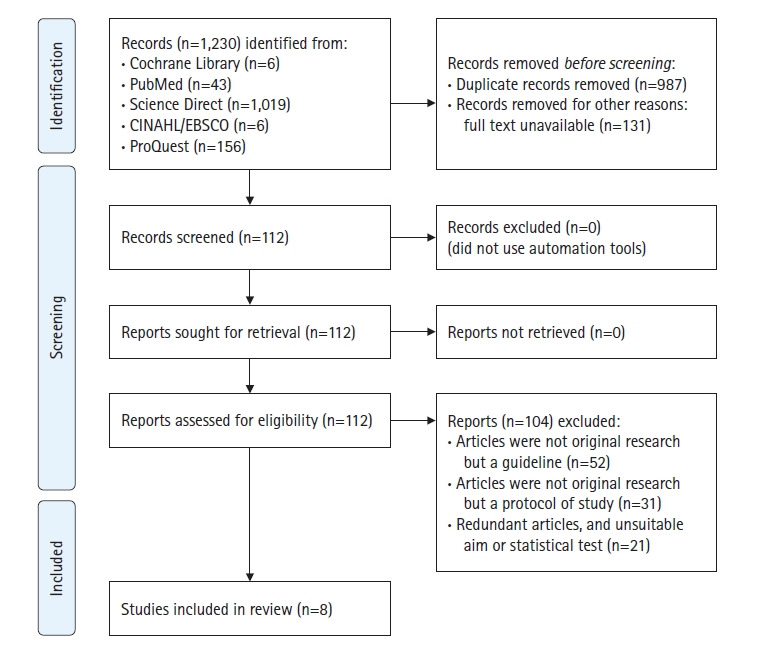
PRISMA 2020 diagram of study selection.

We used the following keywords: “preconception” AND “content” AND “adult(s) OR adolescent(s)” in the following electronic databases: Cochrane Library, PubMed, Science Direct, CINAHL/EBSCO, and ProQuest. The researchers set several inclusion criteria for the selection of research articles. The criteria stipulated that (1) the type of research was qualitative, quantitative, or mixed-methods; (2) the participants in the included studies were predominantly female adolescents and or reproductive-aged adults (aged 15–49 years) following the World Health Organization (WHO) standard for PCC target groups [2]; (3) the studies were published in English; (4) the studies were published within the past decade (January 2012 to March 2022); and (5) the outcomes included PCC knowledge and information. Studies on participants with preexisting conditions, such as chronic illnesses, were excluded if no full text was available. With these keywords, we found 1,230 articles in April 2022. After deleting 987 duplicates and 131 non–full-text articles, 112 articles were selected and read. During the reading, 104 articles were excluded as they did not meet the article selection criteria (e.g., guidelines, protocol articles) (Figure 1). Finally, eight studies [12-19] were included in this review study.

### Methodological quality appraisal

The 16-item Quality Assessment Tool for Studies with Diverse Designs (QATSDD) by Sirriyeh et al. [20] was used to scrutinize the quality of the selected studies. The reliability and validity of the papers were checked, and rigor was evaluated when choosing these qualitative and quantitative studies. All items of the QATSDD can be used for mixed-method research studies, while 14 items can be used for the quality assessment of quantitative and or qualitative studies. Each included paper was checked individually and assigned a score of 0 (=not at all), 2 (=very slightly), 3 (=moderately), or 4 (=complete) according to the identified criteria. The quality appraisal was presented as a percentage of the maximum possible score (42 for both qualitative and quantitative studies) and was generated from the scores given and agreed upon by the first and second authors to establish interrater reliability. A higher score indicated better quality of the paper. Any disagreements about the percentage were resolved through discussion, and when necessary, the third and fourth authors were involved in resolving the disagreement. The assessment results are presented in Table 1.

### Data extraction and synthesis

To summarize the results in a logical way that aligns with the aim of this review, we used a chart to extract key information on the selected articles, including the author’s name, the year and country of the study, the study type and sample, the study’s aim, the article’s quality score by QATSDD, and thematic findings (Table 1). Next, the review was structured by transforming the qualitative or quantitative data from the eligible studies into integrated themes. In the synthesis phase, the authors generated broader themes by reviewing, comparing, and contrasting the different perspectives regarding PCC knowledge and modes of information delivery from the eight selected articles. The integrated themes were formulated to respond to the aims of this scoping review [21].

## Results

### Characteristics of selected studies

As can be seen in Table 1, all included articles were from 2018 and beyond, and four were from 2021. Of the eight selected studies, five were conducted in low- to middle-income countries (Ethiopia, Indonesia, India, and Nigeria) [13,15,17-19], and three were from high-income countries (the United States, Japan, and Australia) [12,14,16]. These findings indicated the increased interest in PCC research following a better understanding of PCC’s importance for women’s and children’s health across the globe. Seven studies were quantitative, including one randomized controlled trial (RCT) [12], one quasi-experimental study [13], one longitudinal intervention [14], and four cross-sectional studies [15-18], while there was one descriptive qualitative study [19]. The studies involved 2,023 female adolescents and reproductive-aged adults attending health care services or at community sites.

### Preconception care knowledge

As noted in Table 1, all studies in this review described some levels and types of basic knowledge regarding PCC, and the details were closely related to the country’s income classification. For instance, in low- and middle-income countries, such as Nigeria, only a few women had good PCC knowledge [18]. Although research in Ethiopia showed that many pregnant women understood human immunodeficiency virus screening, family planning, and hypertension screening, only a few were aware of folic acid and iron consumption and hepatitis B screening, as PCC information was usually provided by health workers only during antenatal visits [17]. A qualitative study in India also reported that of 76 women (aged 15 to 39 years) who participated in focus group discussions, most were well acquainted with the basic concepts of PCC, such as nutrition pre-pregnancy and the effects of tobacco and alcohol effects, but only a few were aware of the need of good mental health, PCC programs, sexually transmitted infections, and the effect of chronic medical conditions; furthermore, 50% had an unplanned pregnancy and most did not access PCC services [19].

On the contrary, research in the United States, as a high-income country, reported that half of the young women had basic PCC knowledge, including treatment goals, the urgency of care, folic acid supplementation, genetic counseling, iron supplementation, and family planning [12]. Meanwhile, an Australian study stated that the adolescents understood well the general health information related to PCC and pregnancy planning [16]. Last, a study in Japan indicated that the female respondents had broader knowledge about PCC, as the study also mentioned cancer screening, infertility, and rubella prevention [14]. These studies indicate that adolescent girls and women in higher-income countries are equipped with more extensive information on PCC.

### Modes of preconception care knowledge information delivery and effectiveness

The prevailing method utilized in providing PCC information was direct health promotion through face-to-face meetings with the female respondents; this method was described in four studies [13-15,18]. For example, a PCC information session delivered through a conventional face-to-face individual health education in the Office of Religious Affairs in Indonesia demonstrated significant effectiveness in changing women’s knowledge regarding physical health, nutrition, and lifestyle [13]. Meanwhile, PCC information group seminars in Japan were conducted through face-to-face individual education, which effectively improved women’s knowledge of preconception nutrition, lifestyle, and physical health [14].

In addition to documenting more extensive PCC knowledge, attitudes, and practices, two studies conducted in high-income countries [12,16] reported advanced modes of PCC information delivery. Those studies reported that PCC information was delivered through digital technology, including web-based PCC educational media [12] and social media (Facebook, Instagram, Snapchat, etc.) [15,16]. An RCT in the United States reported that young women who received an automated PCC intervention from embodied web-based PCC counselors for 12 months showed an enthusiastic attitude toward accessing information that increased their PCC knowledge [12]. Adolescent girls and women utilized internet applications as a source of information related to PCC, and most shared PCC information with others [12,16]. Furthermore, some research indicated that healthcare workers played minimal roles as the source of PCC information for women in Nigeria and Ethiopia [15,18], and PCC services provided by healthcare providers might encourage women’s involvement in PCC, including screening for risk factors and genetic disorders [14,22].

## Discussion

The primary PCC contents identified in this review were mostly consistent with the concept of PCC as explained by the WHO [2]. The reviewed studies involving adolescent girls and women described how information related to PCC has been developed to cover early pregnancy prevention and preconception education, with topics including nutritional supplementation, stress reduction, the dangers of unprepared and young-age pregnancy, and contraception use until the planned time of pregnancy through digital and non-digital education [12,13,16]. However, no studies mentioned the importance of protecting prepregnancy adolescent girls and women from environmental, household, and vehicle exposures, which could be potentially toxic to the pregnancy [2]. In congruence with this finding, the WHO has emphasized that successful PCC comprises critical information on mental health preparedness for pregnancy [1].

This scoping review highlights the importance of PCC information delivery modes in improving PCC awareness among adolescent girls and women in a global world. Individual or in-group conventional face-to-face education was more frequently conducted in low- to middle-income countries [13,18], while digital modes were effectively used to increase PCC knowledge in developed countries [15,16]. In line with these findings, other studies have also suggested that it is effective to deliver other reproductive information not only through conventional educational methods but also by using social media to disseminate content created under WHO guidelines or employing other digital approaches to increase PCC coverage [1,9,10].

This review found that the level of expertise regarding PCC among adolescents and reproductive-aged women in the African and Asian countries where the included studies were conducted was influenced by the educational status of respondents and their partners [17,18], education from health providers [13,14,17], the frequency of antenatal visits, and the history of previous clinic visits [18]. Adolescent girls and women from low- to middle-income countries were less exposed to PCC information than those in high-income countries, who have taken advantage of technological advances, such as web-based and social media, to seek PCC information. This implies the importance of providing a full package of PCC information to wider community groups, such as high school or university students, through multiple educational methods and media. Modified forms of PCC information delivery should also be developed to match the needs of all women [2,23] by taking into account factors influencing PCC delivery, such as local culture, education levels, and the socioeconomic status of women [24,25].

A limitation of this scoping review may have been that we did not supplement the search results with a hand search of the reference lists of the selected studies; thus, we may have missed some relevant studies. Moreover, our inclusion criteria were restricted to articles published in English with available full texts, which may have caused language bias and reduced the number of selected articles to be reviewed. Hence, expanding the selection of studies through a combination of quantitative and qualitative factors could have improved the comprehensiveness of the study.

In conclusion, this review found that although adequate knowledge of PCC is crucial for improving attitudes and practices toward pregnancy preparation, most adolescent girls and women, predominantly in low-to-middle-income countries, lack exposure to more extensive PCC topics, such as pre-pregnancy vaccination, screening, and supplementation. Face-to-face health education by healthcare workers was the predominant mode of PCC information delivery in most developing countries. Since not all women can take advantage of social media, e-Health, and other digital health forums that require modern technology, intensive individual face-to-face health education combined with discussion, continuing feedback, and adequate support may be a good solution to deliver PCC information to reproductive-age women in low- to middle-income countries. Meanwhile, for middle- to high-income countries and other contexts where young women might have widespread access to technology, this study suggests the optimization of digital technology for delivering PCC information.

In future studies, a combination of promotive and educative methods ranging from direct/conventional information delivery methods to digitalization (web and social media platforms) should be used and studied, with a particular focus on the inclusion of specific high-risk groups such as adult women with impaired immunity, infection, and diagnoses of certain serious diseases such as malignancy/reproductive tract diseases. Empirical studies can also be developed further to use a combination of multiple media and educational approaches (personal, in-group, face-to-face, or electronic methods), providing information related to PCC and evaluating knowledge, attitude, and practice adjustments after receiving this information.

## Figures and Tables

**Table 1.** Description of the selected studies (N=8)

First author [Ref]		Year	Country	Study type, sample	Study aims	Quality score, QATSDD	PCC knowledge	PCC information delivery modes and effectiveness
Quantitative studies								
	Bickmore et al. [12]	2020	United States	A randomized controlled trial, 79 women aged 18–25 years (intervention group)	To examine a web-based PCC intervention (“Gabby”)	90%	Increased health literacy about family planning, physical activity, weight management, immunization, drug abuse, infectious and chronic disease care, and folic acid supplementation	The web-based application of PCC conducted by virtual face-to-face individual modes was effective for improving women’s PCC knowledge
	Priani et al. [13]	2019	Indonesia	A quasi-experimental study, 46 unmarried women (intervention group)	To identify the effectiveness of preconception education for women in preparing for pregnancy	74%	Improved knowledge of physical health, nutrition, and preconception lifestyle after an intervention	Training on PCC through face-to-face individual education was effective in improving women’s knowledge of preconception nutrition, lifestyle, and physical health
	Nagusa and Sasaki [14]	2019	Japan	Longitudinal intervention study, 84 mature female workers (20–35 years)	To implement a health education program on PCC	76%	High knowledge about the definition of PCC, eating foods containing folic acid, healthy lifestyle, self-efficacy, stress management, sexually transmitted disease prevention, cervical cancer screening, breast cancer screening, vaccinations, body weight, infertility, and rubella prevention	PCC education through a face-to-face seminar in small groups, group discussion, and feedback was effective in increasing PCC awareness and behaviors. The seminar was combined with a ‘rubella prevention’ video viewing
	Setegn [15]	2021	Ethiopia	A community-based cross-sectional study, of 427 reproductive-age women (15–49 years)	To determine the intention to use and its predictors of PCC use among reproductive-age women	80%	Insufficient knowledge of STI, hypertension, diabetes mellitus screening, folic acid importance before pregnancy, iron intake, and good nutrition	Health workers and school participated in face-to-face education, web-based education, and face-to-face information from family/friends
	Skouteris and Savaglio [16]	2021	Australia	A cross-sectional study, 91 women aged 18–25 years	To examine the proportion, type, and frequency of social media use to seek general health, preconception, and pregnancy-related information or advice	79%	General health, preconception and pregnancy-related health information, and pregnancy planning	Planning a pregnancy was associated with using social media platforms, primarily Facebook, Instagram, and Snapchat for preconception and pregnancy-related health information
	Teshome et al. [17]	2020	Ethiopia	A community-based cross-sectional study, 623 pregnant women (15–49 years)	To assess the knowledge of PCC and associated factors among pregnant women	88%	Most knew about HIV screening, family planning, and hypertension screening, but only a few understood folic acid consumption, iron, and hepatitis B screening as PCC	Not stated
	Ekem et al. [18]	2018	Nigeria	A cross-sectional study, 453 pregnant women (15–44 years)	To assess the level of awareness and utilization of PCC services	85%	Fewer than 50% understood folic acid supplementation, smoking cessation, alcohol cessation, weight control, blood sugar control, hypertension control, and HIV screening	PCC information was delivered by care providers through face-to-face mode
Qualitative studies								
	Doke et al. [19]	2021	India	A qualitative FGD method, 76 women (15–39 years)	To assess women’s basic perceptions, knowledge, and attitudes toward PCC	85%	Insufficient knowledge about pregnancy planning, women’s age, women’s height and weight, daily physical activity, nutrition, smoking, and alcohol consumption, pre-pregnancy medical care, and preconception services	Not stated

FGD: Focus group discussion; HIV, human immunodeficiency virus; PCC: preconception care; QATSDD: the 16-item Quality Assessment Tool for Studies with Diverse Designs.
